# Reactive oxygen species in biological systems: Pathways, associated diseases, and potential inhibitors—A review

**DOI:** 10.1002/fsn3.3784

**Published:** 2023-12-01

**Authors:** Abdur Rauf, Anees Ahmed Khalil, Samir Awadallah, Shahid Ali Khan, Tareq Abu‐Izneid, Muhammad Kamran, Hassan A. Hemeg, Mohammad S. Mubarak, Ahood Khalid, Polrat Wilairatana

**Affiliations:** ^1^ Department of Chemistry University of Swabi Anbar Pakistan; ^2^ University Institute of Diet and Nutritional Sciences, Faculty of Allied Health Sciences The University of Lahore Lahore Pakistan; ^3^ Department of Medical Lab Sciences, Faculty of Allied Medical Sciences Zarqa University Zarqa Jordan; ^4^ Department of Chemistry, School of Natural Sciences National University of Science and Technology (NUST) Islamabad Pakistan; ^5^ Pharmaceutical Sciences, College of Pharmacy Al Ain University Al Ain, Abu Dhabi UAE; ^6^ H. E. J. Research Institute of Chemistry, International Center for Chemical and Biological Sciences University of Karachi Karachi Pakistan; ^7^ Department of Medical Laboratory Technology, College of Applied Medical Sciences Taibah University Al‐Medinah Al‐Monawara Saudi Arabia; ^8^ Department of Chemistry The University of Jordan Amman Jordan; ^9^ Department of Clinical Tropical Medicine, Faculty of Tropical Medicine Mahidol University Bangkok Thailand

**Keywords:** auto‐oxidation, neurodegenerative diseases, redox homeostasis, ROS inhibitors, ROS pathways

## Abstract

Reactive oxygen species (ROS) are produced under normal physiological conditions and may have beneficial and harmful effects on biological systems. ROS are involved in many physiological processes such as differentiation, proliferation, necrosis, autophagy, and apoptosis by acting as signaling molecules or regulators of transcription factors. In this case, maintaining proper cellular ROS levels is known as redox homeostasis. Oxidative stress occurs because of the imbalance between the production of ROS and antioxidant defenses. Sources of ROS include the mitochondria, auto‐oxidation of glucose, and enzymatic pathways such as nicotinamide adenine dinucleotide phosphate reduced (NAD[P]H) oxidase. The possible ROS pathways are NF‐κB, MAPKs, PI3K‐Akt, and the Keap1‐Nrf2‐ARE signaling pathway. This review covers the literature pertaining to the possible ROS pathways and strategies to inhibit them. Additionally, this review summarizes the literature related to finding ROS inhibitors.

## Graphical abstract

Oxidative stress occurs because of the imbalance between the production of ROS and antioxidant defenses. Sources of ROS include the mitochondria, auto‐oxidation of glucose, and enzymatic pathways such as nicotinamide adenine dinucleotide phosphate reduced (NAD[P]H) oxidase. The possible ROS pathways are NF‐κB, MAPKs, PI3K‐Akt, and the Keap1‐Nrf2‐ARE signaling pathway. Various diseases associated with ROS imbalance and types of ROS inhibitors are also discussed in this manuscript.

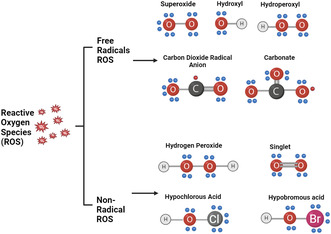

## 1 INTRODUCTION

**FIGURE 1:**
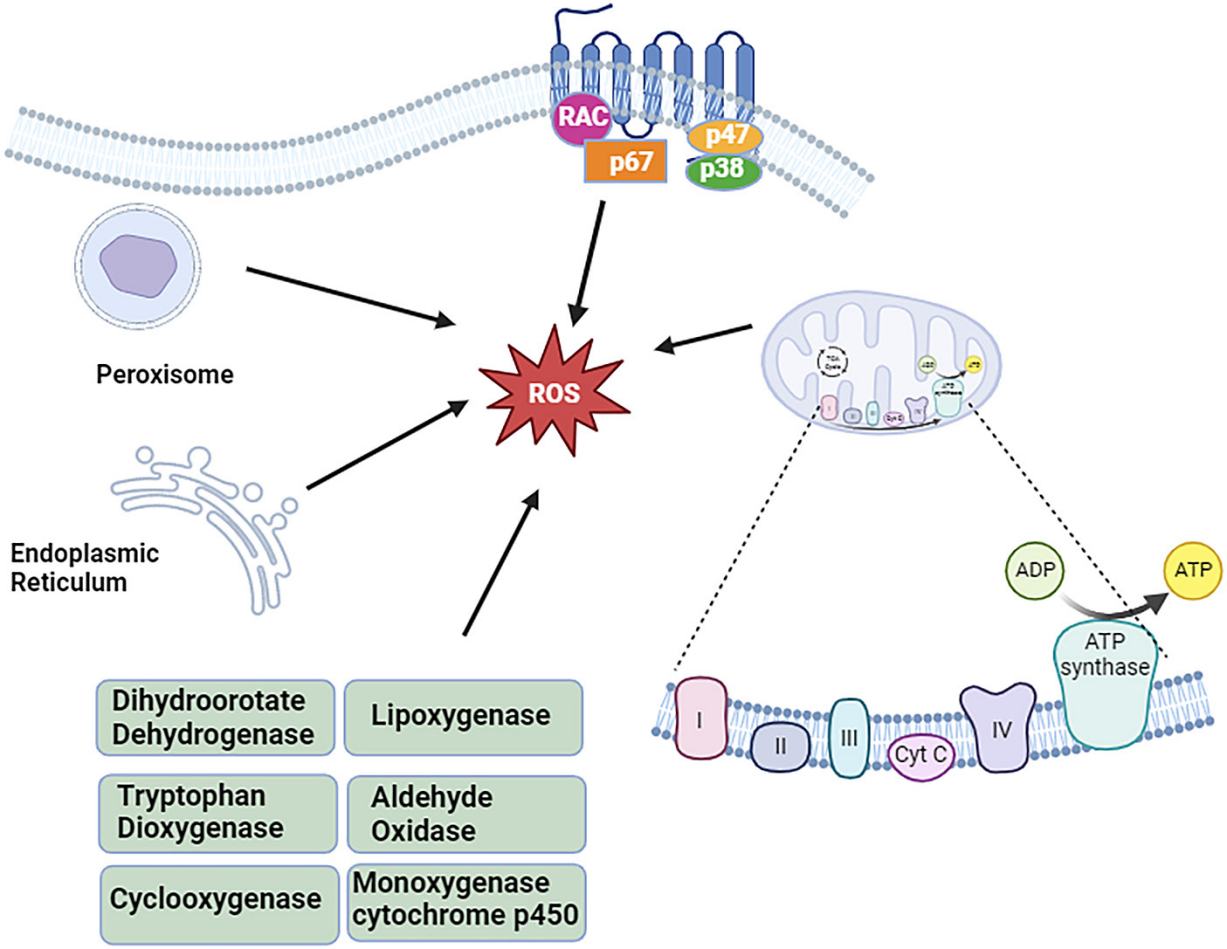
Graphical representation of significant sites for the production of endogenous reactive oxygen species.

Reactive oxygen species (ROS) are non‐radical derivatives of oxygen and chemically reactive oxygen radicals (Halliwell, 2006). ROS are species containing a minimum of one oxygen atom in each molecule but possess enhanced activity compared with molecular oxygen. ROS include free radical species (singlet oxygen, hydroxyl radical, and superoxide) and non‐radical species (hydrogen peroxide H_2_O_2_) (Graphical Abstract) (; Zhang, Cheng, et al., 2016; Zhang, Wang, et al., 2016; Zhang, Zhou, et al., 2016). Both enzymatic and non‐enzymatic reactions result in the constant production of ROS (Pelicano et al., 2004). Typically, physiologically, ROS are generated as a byproduct of normal cellular metabolism occurring in oxidative reaction processes of the mitochondrial respiratory chain (Nathan & Cunningham‐Bussel, 2013). In addition, ROS are generated via various intra‐ and extracellular functions involved in regulating homeostasis in a body, such as cell division, differentiation, and death (Sena & Chandel, 2012; Zhang et al., 2013). Elevated content of ROS results in deterioration of cellular components or activates specified signaling pathways involved in the removal of ROS prior to their causative effect on cell damage (Ban et al., 2021). Furthermore, mitochondria are the primary cellular source for generating ROS, as shown in Figure 1. Most ROS are produced in the electron transport chain (ETC) present in the inner mitochondrial membrane via OXPHOS (oxidative phosphorylation), producing adenosine triphosphate (ATP). Electrons are released by nicotinamide adenine dinucleotide (NADH) and flavin adenine dinucleotide.

Flavin adenine dinucleotide hydride (FADH) produced during the tricarboxylic acid cycle (TAC) is transferred to ETC, which forms water by reducing oxygen molecules under normal conditions. Nevertheless, ROS are formed when this reduction is incomplete and forms a superoxide anion as an end product instead of water. On the other hand, a superoxide anion is formed due to a single electron reduction of oxygen when an electron escapes the ETC process. Other than mitochondria, various other sources of ROS production have been reported in the literature (Sena & Chandel, 2012). These potential sources include nicotinamide adenine dinucleotide phosphate (NADPH) oxidases (NOX), xanthine oxidase (XO), nitric oxide synthase (NOS), and cyclooxygenases (COXs) (Bhattacharyya et al., 2014).

Suitable amounts of ROS have beneficial effects such as wound healing, repairing processes, and killing invading pathogens (Bhattacharyya et al., 2014). In this respect, the balance between the natural antioxidant defense system and ROS generation disturbs a series of events that deregulate cellular functionality, leading to different pathological conditions for almost all vital organs (Flores, 1997). When ROS generation is increased, it disrupts redox homeostasis and induces oxidative stress conditions. ROS impairs the functionality of critical biomolecules and organelles like DNA, proteins, and lipids, leading to various diseases such as carcinogenesis (Ziech et al., 2011), diabetes (Hur et al., 2010), neurodegeneration (de Vries et al., 2008), aging (Vigneron & Vousden, 2010), Alzheimer's disease (AD) (Butterfield et al., 2001), Parkinson's disease (PD) (Onyango, 2008), prion disease (Kim, Choi, et al., 2001; Kim, Rhim, et al., 2001), protein misfolding diseases (Tabner et al., 2005), and ophthalmological diseases (Tezel, 2006). In this context, several naturals (glutathione peroxidase [GPx], Vitamin E, superoxide dismutase [SOD]) and synthetic antioxidants (butylated hydroxyanisole and butylated hydroxytoluene) have been studied for their ROS scavenging activity (Zhang et al., 2009). Based on the preceding discussion, the present work focuses on natural and synthetic ROS inhibitors. This includes ROS pathways and methods of inhibiting them. To achieve this objective, recent relevant references have been obtained from various databases such as (Google Scholar, PubMed), Science Direct, Scopus, and SciFinder, among others. We believe this review will be a valuable addition to the field and a great help to researchers. Further studies are needed to understand the potential of synthetic and natural compounds in inhibiting cellular injury caused by ROS and in treating various associated diseases (He et al., 2017).

## 2 ROS PATHWAYS

Below are some of the important ROS pathways.

### 2.1 The nuclear factor‐Kappa B (NF‐κB)

NF‐κB, a transcription factor, is essential in various cellular processes, such as cell adhesion, immunity, inflammation, differentiation, apoptosis, autophagy, proliferation, and aging (Bonizzi & Karin, 2004). In addition, NF‐κB is responsible for regulating pro‐inflammatory cytokines and can regulate inflammation linked to extensive H_2_O_2_ production. Similarly, disruption of NF‐κB is found to be responsible for various diseases, including asthma, cancer, heart disease, rheumatoid arthritis (RA), neurodegenerative ailments, and inflammation (Baldwin, 2012). The NF‐κB family contains p52/p100 (NF‐κB2), RelA (p65), RelB, p50/p105 (NF‐κB1), and Rel (c‐Rel). The NF‐κB is activated through two distinctive pathways, canonical and non‐canonical. The canonical pathway is initiated due to the production of viruses, stress, and cytokines that cause inflammation, depending on phosphorylation. NF‐κB enters the nucleus, where it activates targeted genetic transcription after proteasomal degradation of IκBa (Devin et al., 2000; Gloire & Piette, 2009). In contrast, the non‐canonical NF‐κB pathway is activated by the B‐cell activating factor (BAFF) (Gardam & Brink, 2014), lymphotoxin β (LTβ) (Bauer et al., 2012), cluster of differentiation 40 (CD40) ligand (Elmetwali et al., 2014), cluster of differentiation 27 (CD27) ligand (Ramakrishnan et al., 2004), human T‐cell leukemia virus (HTLV) (Scherz‐Shouval et al., 2007), and Epstein–Barr virus (EBV) (Zhang, Cheng, et al., 2016; Zhang, Wang, et al., 2016; Zhang, Zhou, et al., 2016). Furthermore, it depends on IKKα and results in the activation of NF‐κB2/RelB complexes by inducing the proteolytic processing of the NF‐κB2/p100 precursor. To maintain a balance between pro‐inflammatory processes and the cells' antioxidative conditions, NF *κ*B‐ and NRF2‐signaling pathways must be well‐coordinated (Pajares et al., 2018).

Literature shows a complex and extensive relationship among ROS and the NF‐κB pathway. Evidently, ROS are known to both inhibit and activate NF‐κB‐mediated transcription, specifically depending upon the type of cell or the intracellular locality of ROS (Hayes et al., 2020). Initiation of the NF‐κB pathway through the triggering of ROS includes the ROS‐dependent phosphorylation of p65 by PKAc, which is essential for its engagement with CBP/300 and its subsequent transcriptional functionality (Hayes et al., 2020). Furthermore, external ROS, particularly H_2_O_2_, can stimulate NF‐κB activation by prompting a distinct phosphorylation of IkBα. This modification subsequently results in the degradation of IkBα within the proteasome. Subsequently, resultant degradation enables dimerized NF‐κB proteins to relocate to the nucleus, facilitating the initiation of transcription for their designated target genes (Sarmiento‐Salinas et al., 2021). Evidence suggests that H_2_O_2_ can enhance the dimerization of IKKγ/NEMO and participate in the phosphorylation‐mediated activation of the IKKβ subunit. This implies the potential inhibition of an IKK phosphatase or the direct oxidation of IKKβ leading to its deactivation and the subsequent activation of the NF‐κB pathway (Hayes et al., 2020).

ROS aid in the regulation of the NF‐κB cascade both through potential activation of NF‐κB in the early phase and inhibition of NF‐κB activation in the late phase. Phosphorylation and consequent IκBα degradation are considered important mechanisms for the ROS‐activated NF‐κB pathway. Additionally, ROS targets both IKK and mitogen‐activated protein kinase kinase 1 (MEKK1) and deactivates their functionality via S‐glutathionylation, therefore influencing the activity of NF‐κB. On the other hand, the activity of NIK (NF‐κB inducing kinase) is regulated by ROS via oxidation of cysteine residues and phosphatase inhibition (Khan et al., 2021).

### 2.2 MAPKs pathway

Protein kinase (MAPK) is activated by mitogen cascades, which include offline cell‐related kinases (ERK1/2), c‐Jun N‐terminal kinases (JNK), p38 kinase (p38), and the significant MAP kinase 1 pathway (BMK1/ERK5). Structural and functional homology has been observed in various members of the MAPK group. In this regard, several investigations to identify the components that target different MAPK pathways have been reported (Fan et al., 2019). Major intracellular signal transmission mechanisms play a vital role in cellular processes like cell division, differentiation, and death (Junttila et al., 2008). Likewise, a proline residue directs all serine/threonine kinases (BMK1, p38, JNK, and ERK). Moreover, MAPK‐cascade pathways include three kinases: mitogen‐activated protein kinases (MAPKKKs), mitogen‐activated protein kinase kinases (MAPKKs), and mitogen‐activated protein kinases (MAPKs). These pathways result in the regulation of different cellular functionalities (Chen et al., 2001; Pimienta & Pascual, 2007). On the other hand, JNK‐pathway, Apoptosis signal‐regulating kinase 1 (ASK‐1), an initial protein‐is also responsible for p38‐pathway activation. Oxidative stress has also been found to directly or indirectly influence ASK1, mitogen‐activated protein kinases kinase 1–4 (MEKK1‐4), and mixed‐lineage protein kinase‐3 (MLK3), hence successively activating the p38 pathway (Cuadrado & Nebreda, 2010; Shin et al., 2005).

Evidence indicates that ROS may oxidize and inactive MAPK phosphatases. Concurrently, they trigger the activation of signaling through the platelet‐derived growth factor receptors (PDGFR) and epidermal growth factor receptor (EGFR), independent of ligand binding via the mediation of the ERK and RAS pathways (Aggarwal et al., 2019; Lee et al., 2018; Sidhanth et al., 2018). Various studies have reported H_2_O_2_ as a vital mediator for ligand‐independent phosphorylation of RTKs (receptor tyrosine kinases) (Kanojia et al., 2017). The estrogen metabolism within breast carcinoma leads to the generation of H_2_O_2_, which results in the activation of ERK1/2. Activated ERK1/2 contributes to cellular proliferation and enhanced survival. Elevated ROS are linked to cell cycle arrest, senescence, and the induction of apoptotic conditions via the ASK1/JNK/p38 signaling cascade (Kirtonia et al., 2020). Apoptosis signal‐regulated kinase‐1 (ASK‐1) and reduced thioredoxin (TRX) result in a complex formation leading to the inactivation of ASK‐1. During oxidative stress conditions, hydrogen peroxide oxidizes the cysteine residue thioredoxin, which results in dissociated ASK‐1 for JNK activation and a p38 cascade leading to the induction of an apoptotic condition (Kirtonia et al., 2020).

### 2.3 PI3K‐Akt signaling pathway

Phosphoinositide‐3‐kinase (PI3K) pathways are involved in various vital cellular activities, such as cell cycle progression, propagation, induction of apoptotic conditions, autophagy, protein synthesis, drug resistance in response to cytokines (IL2, IL6, and IL17), growth factors [Vascular Endothelial Growth Factor (VEGF), nerve growth factor (NGF), Platelet‐derived growth factor (PDGF), and Epidermal growth factor (EGF)], and hormones (prostaglandin: PGE2) (Chen et al., 2001; Innocenti et al., 2003; Pimienta & Pascual, 2007; Qiu et al., 2014). The binding factor present in the growth factor receptors aids in the direct stimulation of class 1A PI3K that is bound by its regulatory unit or insulin receptor‐like adapters. Insulin receptor substrate (IRS) proteins result in the activation of PI3K. After that, activated PI3K updates the compilation of phosphatidylinositol 3,4,5‐triphosphate (PIP3) from phosphatidylinositol 4,5‐bisphosphate (PIP2). This procedure includes the activation of phosphoinositide‐dependent protein kinase (PDK) and protein kinase B (Act), which results in improving the activity and regulation of p53, BAD, Forkhead box O (FOXO), glycogen synthase kinase‐3 (GSK3), and mTOR1 (Cohen & Frame, 2001; Zhang et al., 2014; Zhang, Gan, et al., 2011; Zhang, Lau, & Monks, 2011). In this respect, ROS directly activate PI3K and downregulate PTEN (phosphatase and tensin homolog), responsible for negative regulation of PIP3 synthesis, hence suppressing Akt activation through the oxidation of cysteine residues inside the active center (Innocenti et al., 2003; Leslie & Downes, 2002).

### 2.4 Keap1‐Nrf2‐ARE signaling pathway

Keap1‐Nrf2‐ARE plays a crucial role in maintaining redox cellular balance and metabolism. It additionally promotes oxidative stress, leading to various serious illnesses, including diabetes, cancer, PD, and AD. The Nrf2 is characterized as an important transcription factor that regulates the expression of antioxidative proteins, therefore protecting against oxidative damage (Xu et al., 2023). This signaling pathway contains three cell components: Kelch‐like ECH‐associated protein 1 (Keap1), nuclear factor erythroid 2‐related factor 2 (Nrf2), and antioxidant reactions (ARE) (Zhang, Cheng, et al., 2016; Zhang, Wang, et al., 2016; Zhang, Zhou, et al., 2016). KEAP1 is the main sensor protein for electrophilic and oxidative stresses, and it also functions as an NRF2 inhibitor and harbors various cysteine residues leading to oxidation. In this respect, Cys151 disulfide formation in the KEAP1 dimer results in conformational alteration of KEAP1 that prevents the ubiquitination of NRF2, thus serving as a transcription factor in expressing antioxidative defense proteins. Multiple cysteine thiol groups of KEAP1 respond selectively to different oxidants. The sirtuin family of deacetylases and thioredoxin reductase 1 regulates the NRF2–KEAP1 system. Moreover, redox‐sensitive microRNAs aid in modulating the NRF2 system (Sies & Jones, 2020).

Persistent NRF2 activation has been documented in various human cancers, such as skin, breast, lung, prostate, and ovarian cancers (Almeida et al., 2020; De la Vega Rojo et al., 2018; Kerins & Ooi, 2018). Mutated NRF2, KEAP1, and predominant oncogenes like Myc and KRAS are reported to trigger NRF2 activation. Irregularities within the NRF2–KEAP1 pathway have been documented in metastasis, genomic instability, drug resistance, apoptotic resistance, and alterations in cellular metabolism in various cancer cells (de la Vega Rojo et al., 2018; Kerins & Ooi, 2018) Depleted NRF2 showed reduced tumor growth through enhancement of oxidative stress‐dependent cellular death (Kirtonia et al., 2020). During normal physiological circumstances, NRF2 experiences proteasomal degradation as a result of its capacity to engage with KEAP1 (Kelch‐like ECH‐associated protein 1) together with the Cullin 3 (Cul3) E3 ubiquitin ligase (Kirtonia et al., 2020). Conversely, induced oxidative stress elevates ROS levels, oxidizes KEAP1, and obstructs the interaction between NRF2 and the KEAP1 degradation complex (Kirtonia et al., 2020).

## 3 ROS SIGNALING, TRANSDUCTION AND CELLULAR PROCESS

### 3.1 Autophagy

Autophagy is a tightly controlled cellular breakdown system that uses lysosomes to break down protein aggregates, impaired organelles, and invading microbes. Autophagy dysfunction has been linked to various human disorders, including cancer, diabetes, and cardiovascular disease. Under stressful circumstances like hunger, endoplasmic reticulum (ER) stress, organelle destruction, and pathogen infection, autophagy can be initiated. The production of ROS has become a key element in the activation of autophagy. Oxidative stress is caused by the accumulation of H_2_O_2_ in the cell. ATG4 is an autophagy gene that plays a role in the autophagic process. During hunger, ATG4 is a direct target for the oxidation of H_2_O_2_. The ATG4 activity can be oxidized by the accumulation of H_2_O_2_ (Scherz‐Shouval et al., 2007). In mammals, the autophagic process is very complicated, as various regulating mechanisms may function in distinctive cell types and at several conditions for maintaining proteostasis. In short, different signals activate the ULK complex. The activated ULK complex acts on the class III phosphatidylinositol‐3 kinase complex that produces PtdIns3P (phosphatidylinositol‐3‐phosphate) in the phagophore membrane, which helps in recruiting other proteins to the nucleation site (Pajares et al., 2018). For autophagy initiation, oxidized ATG4 stimulates LC3/ATG8 lipidation (Zhang, Cheng, et al., 2016; Zhang, Wang, et al., 2016; Zhang, Zhou, et al., 2016). LC3‐PE accumulates on autophagosomal membranes in response to ROS, boosting the early stages of the formation of the autophagosomes.

ROS can indirectly control autophagy by activating the MAPK family, which includes JNK, p38, ERK, and p38 (Torres & Forman, 2003). MAPK family members are activated by MAPKKK, MAPKK, and MAPK in a three‐tier kinase cascade (Sakon et al., 2003). JNK activation that lasts for a long time might significantly increase the number of cells in the body. Finally, the p53 pathway is activated by detecting redox stress in the cell, and P53 transactivates many genes as a transcription factor. Autophagy inducers may activate JNK and Sestrin2 bounds with the TSC1/autophagy complex, whereas TSC2 induces TSC2 phosphorylation and activation, which leads to autophagy (Bensaad et al., 2009). Chaperone‐mediated autophagy (CMA) is an autophagy in which soluble proteins are degraded by specific KFERQ‐like motifs. Proteins having this pentapeptide are recognized and attached to chaperone HSC70. Chaperone HSC70 transports substrate proteins to lysosomes, where they interact with the lysosomal receptor (Lysosome‐associated membrane protein‐2, LAMP2A). Furthermore, LAMP2A then multimerizes and produces a translocon that allows the entry of substrate proteins into the lysosome. This is facilitated through lysosomal HSC70 (lysHSC70) and other chaperone groups (Pajares et al., 2018).

### 3.2 Apoptosis

Apoptosis is a programmed series of events that are dependent on energy, along with morphological characteristics like shrinkage of cells, condensation of chromatin, and the occurrence of apoptotic bodies (ApoBDs) without inflammatory reactions. Alternatively, three pathways result in apoptosis: (a) intrinsic pathway, (b) extrinsic pathway, and (c) perforin/granzyme‐induced pathway (Su, Shen, et al., 2019;Su, Zhang, et al., 2019). Cell apoptosis is triggered by intracellular and extracellular signals that pass through death receptors and mitochondrial pathways. When a cell enters apoptosis, ROS levels rise due to disruption of equilibrium in intracellular redox and irreversibly oxidative changes of lipids, proteins, or DNA, thus triggering oxidative stress‐induced apoptotic signaling (Song et al., 2011). ROS is responsible for the induction of apoptotic conditions in cancer cells through a TNF‐related apoptosis‐inducing ligand and upregulation of CD95 and TRAIL‐death receptors by activation of NF‐κB (Izeradjene et al., 2005). JNK belongs to the MAPK family of proteins and appears to play a critical role in mitochondrial malfunction and consequent apoptosis, according to mounting data. In this regard, shikonin is a natural naphthoquinone derivative that helps promote apoptotic conditions in various cancer cells. Research findings showed that apoptotic conditions increased in K526 cells pre‐treated with Shikonin. Moreover, ROS production was accelerated, and JNK and P38 were activated (Mao et al., 2008). ROS/JNK activation can also increase and maintain p53 activity, producing a powerful apoptotic response in cancer cells. In this context, activated ASK1 inhibits JNK activity and causes apoptosis through mitochondrial signaling or pro‐apoptotic gene transcription regulated by AP‐1 (Soga et al., 2012).

### 3.3 Necrosis

Necrosis is a cellular death that differs from apoptosis. Necroptosis generated by TNF is a type of planned necrosis. Because of its high expression, RIP3, a protein kinase, triggers cellular death in numerous cell lines (Oberst et al., 2011). The activity of RIP1 and RIP3 regulates programmed necrosis. Necrosomes trigger a pro‐necrotic signal that requires RIP1 and RIP3 phosphorylation (McQuade et al., 2013). In necrosis‐induced cells, depletion of RIP3 lowers the content of ROS; however, raised RIP3 levels elevate the generation of ROS. Necrosis enhancing impact of RIP3 is mediated by an increase in energy metabolism‐related ROS generation (Izeradjene et al., 2005). Published research indicated that RIP1 and RIP3 phosphorylation inside the pronecrotic complex stabilizes their interaction, triggers the activity of pronecrotic kinase, and initiates ROS generation (Cho et al., 2009). The relationship between STAT3 and GRIM‐19 (the mitochondrial ETC Complex‐I component) may regulate the elevated production of mitochondrial ROS triggered by the activation of RIP1 phosphorylation. In addition, STAT3 interacts with GRIM‐19 in the mitochondria, resulting in increased ROS production and cell death (Kim et al., 2007). Furthermore, direct connections between RIP3 and the enzymes glutamate‐ammonia ligase (GLUL), glutamate dehydrogenase 1 (GLUD1), and glutamate dehydrogenase 2 (GLUD2) have been investigated. GLUD1 and glycogen phosphorylase (PYGL) help improve mitochondrial functionality and energy metabolism (Zhang et al., 2009). In necrosis, the NOX (NADPH oxidase) enzyme is activated on the cellular surface (Linkermann et al., 2013). In contrast, the mixed lineage kinase domain‐like (MLKL) emerges as a main RIP3 downstream component of TNF‐induced necrosis among ROS‐induced necrosis. During TNF‐mediated necrosis, MLKL is responsible for ROS production and late‐phase JNK activation (Sun et al., 2012).

### 3.4 Ferroptosis

Ferroptosis, a programmed cell death, is an iron (Fe)‐dependent elevation in levels of ROS. Ferroptosis plays a vital role in cellular proliferation, senescence, and differentiation. According to published research, iron accumulation in senescent cells results in resistance to ferroptosis. Ferroptosis, a pathological process, causes apoptosis in kidney diseases, stroke, cancer, degenerative ailments, ischemia–reperfusion injuries, and intracerebral hemorrhage in mammals (Wang et al., 2023). Ferrostatin‐1 (Fer‐1), a ferroptosis inhibitor, prevents lipid oxidation and may also prevent cyst development in polycystic kidney disease, resulting in the requirement of ferroptosis for cellular proliferation in fibrosis‐related ailments. *G px4* deletion, a ferroptosis‐executing gene, neuron‐like cells became more sensitive to ferroptosis upon differentiation. The results of this study reinforce the vulnerability of neuronal context to ferroptosis and recommend the value of ferroptosis in neuroprotection (Su, Shen, et al., 2019; Su, Zhang, et al., 2019). Furthermore, ferroptosis is an iron‐dependent, oxidative cell death that various small compounds may cause with different structures, such as RSL3, sulfasalazine, and erastin. Additionally, ferroptosis is caused by a breakdown in the antioxidant system, leading to a loss of cellular redox equilibrium. Apoptosis is not the same as ferroptosis (Yang & Stockwell, 2008).

RSL‐induced cell death lacks the hallmarks of apoptosis, such as mitochondrial cytochrome c release, caspase activation, and chromatin breakage. On the other hand, ferroptosis is associated with higher intracellular ROS levels and an inhibitory impact from genetic inhibition or iron chelation (Yagoda et al., 2007). The release of ROS causes the separation of cells and ferroptosis. Furthermore, deferoxamine, an iron chelator, inhibited ROS accumulation and ferroptosis (Dixon et al., 2012). SLC7A11, an antiporter, imports cystine absorption in the cell, whereas it exchanges with glutamate in the absence of Na^+^ (Sato et al., 2000). SLC7A11 transcriptional repression mediated by p53 is required for ROS‐induced ferroptosis. Cysteine absorption is inhibited by p53, which makes cells more susceptible to ferroptosis (Jiang et al., 2015). Along this line, scientists recently discovered that SAT1 is essential for ferroptosis reactions. In polyamine catabolism, SAT1 is a rate‐limiting enzyme that converts spermine to spermidine and spermidine to putrescine. Lipid peroxidation occurs because of the activation of SAT‐1, making cells more susceptible to ferroptosis in response to ROS‐induced stress (Ou et al., 2016). Ferroptosis is induced via structurally diverse small molecules like RSL3, sulfasalazine, and erastin and could be prevented by using lipophilic antioxidants such as liproxstatins, ferrostatins, Vitamin E, and CoQ10. As mentioned earlier, ferroptosis is triggered by the elevation of ROS, reduction of the antioxidant glutathione (GSH), and increased intracellular iron content, which results in lipid peroxidation and cell death. Several investigations have established that the activity of GPX4 reduces or excessive iron results in ferroptosis (Su, Shen, et al., 2019; Su, Zhang, et al., 2019).

## 4 DISEASES CAUSED BY ROS

### 4.1 Neurodegenerative diseases

**FIGURE 2:**
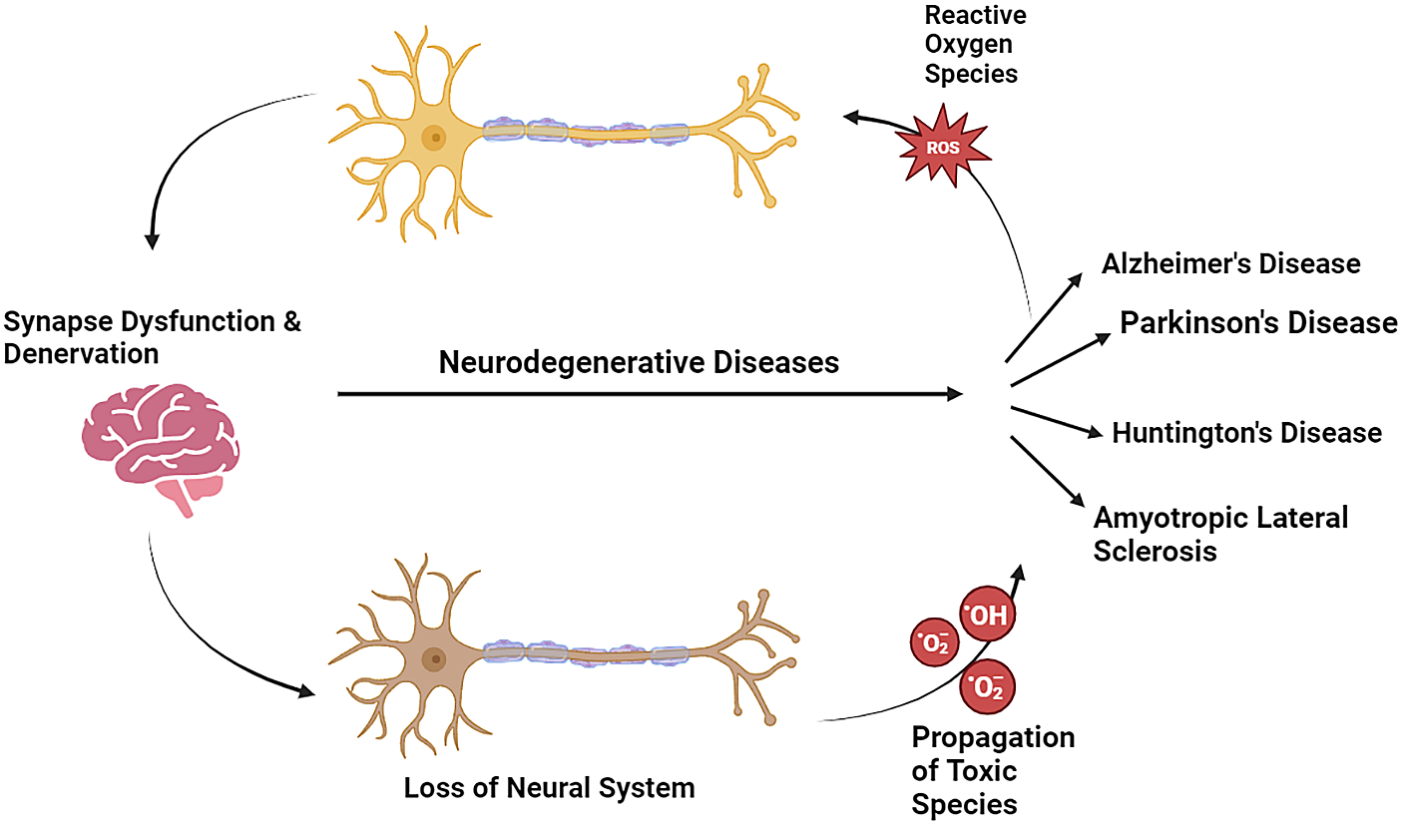
Overview of some neurodegenerative diseases and their associated mechanisms involving neuronal death pathways.

Neurodegenerative diseases are characterized by an increased and pathological loss of neurons, resulting in cognitive impairment, dementia, perturbed motor control, and, eventually, death. Neurodegenerative diseases are of great concern over public health nowadays owing to their incapacitating nature and lack of efficient treatments (Simpson & Oliver, 2020). AD, PD, amyotrophic lateral sclerosis, and multiple sclerosis are examples of neurodegenerative disorders (Figure 2). In these neurodegenerative ailments, the nerve cells present in the brain and spinal cord regions suffer dysfunctionality and excitotoxicity. Lastly, apoptotic conditions arise, resulting in functional and sensory impairment. Since its inception, the metabolic rate of the brain has been great, yet the ability for cellular regeneration is low. Damaged nerve cells in the brain are particularly vulnerable. In this regard, unsaturated lipids make up a significant portion of the biochemical makeup of neurons. Peroxidation and oxidative modification are used to metabolize the substance. Because the brain is not highly resilient and is high in antioxidant defenses, it is particularly vulnerable to ROS (Ou et al., 2016; Rice et al., 2002). Patients with PD, AD, and amyotrophic lateral sclerosis have lipid peroxidation indicators in the brain, hippocampus, and spinal fluid (Butterfield et al., 2002). The protein oxidation marker has been found helpful and elevated in the hippocampus and motor neurons of AD and PD patients, respectively (Aoyama et al., 2000; Miller et al., 2013). Within this context, the DJ‐1 gene is involved in reducing oxidative stress and transcriptional control, and dysfunctionality of the DJ‐1 gene may develop PD. Furthermore, DJ‐1 is an antioxidant with a cytoprotective effect, and mutations in AD‐related genes have been linked to increased oxidative damage and oxidative injury susceptibility (Nunomura et al., 2017).

In AD, uneven ROS production and a diminished amount of ROS‐scavenging enzymes cause oxidative damage to amyloid β‐peptide (Aβ‐peptide). Recently, this association of oxidative damage with ROS and Aβ‐peptide has been reported (Cheignon et al., 2018). In this context, research findings indicated an increase in the concentrations of 4‐hydroxynonenal (4‐HNE) and malondialdehyde in the brains of AD patients. In addition, 4‐HNE results in apoptotic conditions due to the induction of toxicity and alteration in microtubule conformation (Gęgotek & Skrzydlewska, 2019; Xiao et al., 2017). Moreover, in an AD patient's parietal cortex and hippocampus portions, protein oxidation is evident from elevated carbonylated protein contents. ROS induce protein carbonylation and lead to protein cleavage by forming alkoxide radicals. These radicals target proteins involved in ATP synthesis (glyceraldehyde 3‐phosphate dehydrogenase, pyruvate kinase, malate dehydrogenase, and α‐enolase) and glucose metabolism (Butterfield & Boyd‐Kimball, 2019). On the other hand, oxidative stress and ROS are the main factors that cause PD. Increased concentrations of oxidized proteins and lipids have been reported in the PD substantia nigra. In addition, the levels of DNA oxidation biomarkers (8‐hydroxyguanine and 8‐hydroxy‐2′‐deoxyguanosine) were found in high quantities in PD. Furthermore, the level of 4‐HNE protein is directly linked with PD, similar to AD. Production of ROS and oxidation of dopamine are essential in the neurodegenerative mechanism of PD. An increased Fe^2+^ release induces oxidative stress due to overproduced ROS (Kwon et al., 2018; Weng et al., 2018). In other neuro‐related diseases like Huntington's disease (HD), evidence has shown a strong association with ROS‐induced oxidative stress, although the etiological mechanism is still unclear. Early oxidative stress and mitochondrial dysfunction are associated with HD; even this mechanism is still unclear. Elevated 8‐hydroxy‐2′‐deoxyguanosine, a DNA oxidation biomarker, has been reported in blood samples of HD patients coupled with breakage of DNA strands due to ROS production (Khan et al., 2018; Túnez et al., 2011).

Gastrodin, a bioactive constituent in the *Gastrodia elata* rhizome, was reported to have neuroprotective potential in experimented PD models. Research findings showed that gastrodin increases the content of SOD, heme‐oxidase1, NRF2 nuclear translocation, and glutathione (GSH) contents in the PD mice model (Wang et al., 2014). Another study conducted on Tg2576 transgenic mice found that gastrodin enhanced learning and memory ability while also lowering the amount of intracellular ROS. In addition, the expressions of activated PKR, BACE1, and activated eIF2 were enhanced in H_2_O_2_‐stimulated cells and the brains of mice (Zhang, Cheng, et al., 2016; Zhang, Wang, et al., 2016; Zhang, Zhou, et al., 2016).

### 4.2 Inflammatory bowel disease

Inflammatory bowel disease (IBD) is a term that refers to chronic, relapsing inflammatory illnesses such as Crohn's disease and ulcerative colitis. The exact cause of IBD is still unclear; however, it could be caused by a combination of environmental factors, genetics, the immune system, and gut bacteria. These variables interact to cause dysregulated inflammatory responses in the gut as a result of a disturbance of intestinal homeostasis. Because inflammation is closely linked to the generation of reactive intermediates, such as ROS and RNS, oxidative stress has emerged as a possible mechanism explaining IBD pathogenesis. As an evolutionarily conserved reaction to microorganisms or illnesses, the host creates ROS. Controlling oxidative stress may aid in preserving intestinal homeostasis in chronic inflammation and dysbiosis (Chong et al., 2017). ROS can cause inflammation by activating signaling pathways such as extracellular signal‐regulated kinase, protein kinase‐C (PKC), JNK, and RTKs. Furthermore, NF‐B is one of several redox‐sensitive inflammatory transcription factors that cause cellular inflammation (Ballal et al., 2015). On the other hand, chronic oxidative stress causes tissue damage and alters the microbial balance in IBD patients. Some beneficial microorganisms produce various products that aid in the reduction of gastrointestinal damage. However, human clinical studies have revealed diverse results regarding the function of microbes in IBD (Bourreille et al., 2013).

In the gut, overproduction of ROS results in oxidative stress conditions, which enhance cellular damage through modifications in the function of proteins and the peroxidation of lipids. Intestinal epithelial cells (IECs), macrophages, and neutrophils generate nitric oxide and superoxide through NOX and iNOS activation, respectively. Production of reactive oxygen and reactive nitrogen species is elevated in IECs due to the activation of NOX and iNOS. Overproduction of ROS damages cytoskeleton proteins and alters tight junctions and epithelial permeability in IECs, eventually resulting in a disrupted barrier. Oxidative stress aggravates the gastrointestinal tract and initiates IBD (Asakura & Kitahora, 2018; Tian et al., 2017).

### 4.3 Cancer

Increased oxidative stress has been seen in several kinds of cancer cells as compared to normal ones. In tumor cells, ROS serve a variety of activities owing to the shape of the radical, concentration, and position inside the cell DNA damage. Through biological signaling pathways, protein degradation can lead to cancer initiation, which has been demonstrated to be linked to tumor cell proliferation, survival, and development. Cancer cells have a more significant metabolism than normal cells, allowing them to function continually. They must create and maintain greater ROS concentrations to sustain their high proliferation rate. In this respect, ROS generation can induce DNA oxidative damage, leading to mutations. In addition, aging and carcinogenesis are aided by a mutation in DNA (Matsui et al., 2000). On the other hand, ROS and carcinogenesis are linked by IB kinase (IKK), an NF‐B activator. IKK is implicated in chemically induced liver cancer, as evidenced by increased JNK activation, ROS generation, and hepatocyte death in IKK experimental mice. In carcinogenesis mice, the antioxidant treatment effectively prevents the activity of JNK and compensatory cell growth (Luo et al., 2005). In humans, NF‐B is controlled by an intracellular molecule known as TAK1 (Roh et al., 2014). Mutations in TAK1 have been found in individuals suffering from prostate cancer and diffused large B‐cell lymphoma. Furthermore, ROS activates MAPK pathways that are implicated in apoptosis via the activity of JNK, p38‐MAPK signaling proteins, and ERK. Antioxidant‐mediated MAPK modulation is also used to treat heart failure (Martinez et al., 2016).

In different cancers, research findings showed a high content of ROS. Moreover, ROS enhances pro‐tumorigenic signaling, increases cellular proliferation, and induces DNA damage and genetic instability (Moloney & Cotter, 2018). ROS are high‐reactive molecules causing DNA damage and affecting DDR (DNA damage response). Preclinical and clinical evidence has shown that ROS affects the genotoxic stress resulting from chemotherapeutic and radiation candidates. Mechanistically, ROS affects the cell's response to DNA damage resulting from genotoxic therapies, specifically double‐strand breaks (DSBs). This resulted in the clinical assessment of ROS‐modulating agents and genotoxic chemotherapies having a mixed success rate to date. Furthermore, the influence of ROS on DNA damage response and its clinical relevance has also been investigated in the literature (Srinivas et al., 2019). At low contents, ROS works as a signaling agent at low concentrations, aiding in tumorigenesis. In contrast, ROS acts as reactive molecules modulating cancer at elevated concentrations owing to their proapoptotic and detrimental effect on cancer cells (Chio & Tuveson, 2017; de Sá Junior et al., 2017).

The elevated generation of ROS was linked to a disruption in transforming growth factor (TGF) signaling, which resulted in inhibiting the GPx1 antioxidative enzyme (Liu et al., 2008). Along this line, ROS can create DNA damage that stimulates the p53 gene in normal cells, activating it, where DNA repair and stress responses are two examples (Trachootham et al., 2009). Because an increase in ROS in cancer cells may have an impact, inherent oxidative stress plays an essential role in the onset and progression of cancer. On the other hand, excessive ROS generation can be harmful to cancer cells. Cancer cells under high oxidative stress are more likely to die. Other ROS adverse effects caused by external substances make the cells more prone to harm. Therefore, using redox modulation to manipulate ROS levels can be used to selectively suppress cancer cells (Young et al., 2015). In this respect, green tea (*Camellia sinensis*) contains elevated levels of catechins, such as epigallocatechin‐3‐gallate (EGCG). Green tea and EGCG have been found to decrease cancer cell growth in animal models of carcinogenesis. Tea polyphenols are significant radical scavengers owing to the occurrence of di‐and trihydroxy groups. EGCG treatment in experimental mice enhances the expression of the genes gamma‐glutamyltransferase, glutamate cysteine ligase, and hemeoxygenase‐1 (Shen et al., 2005). Human prostate cancer (LNCaP) cells treated with EGCG promote the activation of p53 through acetylation at the Lys382 and Lys373 residues, resulting in a rise in GSTP1 (Thakur et al., 2016). Berberine, a bioactive component having anticancer potential, is present in berberis amurensis (Xie et al., 2009), in addition to EGCG. According to published research, berberine inhibits topoisomerase and telomerase and reduces the growth of many cancers. Berberine can scavenge ROS, decrease lipid peroxidation, and lower metal ion concentrations in lipid peroxidation (Saeidnia & Abdollahi, 2013).

### 4.4 Diabetes

Overproduction of mitochondrial ROS is directly linked to diabetes and its consequences, according to mounting evidence. ROS causes insulin resistance in T2DM and a reduction in the cellular function of the pancreas (Kanikarla‐Marie & Jain, 2015). High glucose levels activate various mitochondrial enzymes, including XO, NO synthases, and NADPH oxidase (Gupta et al., 2002). Oxidative stress causes glucotoxicity and lipotoxicity in β‐cells in diabetes (Poitout & Robertson, 2008), resulting in enhanced toxicity to β‐cells due to the two pathogenic processes of diabetes: hyperglycemia and hyperlipidemia. Both high glucose and fat concentrations have been shown to damage cells in vitro and in vivo studies (Harmon et al., 2001), whereas high blood glucose levels cause oxidative stress that affects the expression and production of insulin mRNA. On the other hand, antioxidant reagents restore insulin promoters and insulin mRNA expression in cultured cells (Yoon et al., 2006). To maintain normal physiological status, cells require a large number of cell signaling pathways, including insulin receptor substrate‐1 (IRS‐1), insulin receptor (IR), insulin or insulin‐like growth factor (IGF‐1), ERK kinases, and PI3K/Akt. When ROS generation surpasses normal physiological levels, those signaling pathways are harmed, resulting in decreased insulin secretion and potentially insulin resistance (Powell et al., 2003). A vast body of data suggests that oxidative stress conditions play a significant role in the pathophysiology of diabetes. In addition, the pancreas has a limited capability for detoxifying ROS, making it vulnerable to ROS‐induced damage. As a result, research into natural or synthetic antioxidants may be extremely valuable in treating diabetes and associated disorders (Panahi et al., 2017).

Overproduction of ROS causes oxidative stress along with various cellular changes. In diabetes, the elevated content of glucose enhances ROS production and induces apoptotic conditions in β‐cells. It has been observed that in the progression of diabetes, the induction of apoptotic conditions and necroptosis plays a significant role (Volpe et al., 2018). The production of ROS is a contributing factor in diabetic‐related vascular complications. ROS production has been reported to worsen in macrophages and monocytes of diabetic patients owing to the shift in glycolytic metabolism. Macrophages are considered key players in promoting inflammation and diabetes progression via the release of proinflammatory cytokines. ROS are critical mediators in the upregulation of proinflammatory cytokines. Hence, the production of ROS induced by a hyperglycemic state might favor the initiation of proinflammatory macrophages in diabetes. ROS induce STAT6, NF‐κB, STAT1, and MAPK signaling and affect macrophage differentiation by reprogramming epigenetics (Rendra et al., 2019). Gallic acid is a significant biologically active polyphenolic with anti‐inflammatory and antioxidant properties (Mansouri et al., 2014). Gallic acid has hypoglycemic and insulin secretagogues activities in streptozotocin (STZ)‐induced diabetic experimental rats, according to growing evidence. In this regard, gallic acid supplementation can repair islet β‐cells, restoring normal insulin levels and reducing oxidative stress (Latha & Daisy, 2011). Moreover, gallic acid enhances glucose absorption by activating and translocating GLUT4 in the PI3K/p‐Akt pathway. Additionally, gallic acid, a partial agonist of PPAR, reduces fat‐rich diet‐fed STZ‐induced experimental type‐2 diabetic rats and improves glucose uptake via translocating and activating GLUT4 in the PI3K/p‐Akt signaling pathway (Gandhi et al., 2014).

## 5 INHIBITION OF ROS

In the past two decades, the discovery of ROS innovative inhibitors has attracted researchers' interest (Ellis et al., 1988). As discussed in previous sections, ROS plays a crucial role in various physiological processes, including cellular apoptosis, proliferation, adhesion, migration, and regulation of the immune response (Chen et al., 2016; Chiu & Dawes, 2012; Choi et al., 2010; Morris et al., 2022; Zhang et al., 2018). However, ROS production must be closely controlled to minimize the negative consequences of a redox imbalance. Elevated levels of ROS have been linked to various pathological conditions such as cancer, cardiovascular complications, neurodegenerative ailments, and diabetes (Bhatia et al., 2020; Moris et al., 2017; Shiota, 2021; Simunkova et al., 2019; Wang, Elksnis, et al., 2018; Wang, Xiao, et al., 2018; Xu et al., 2020). Consequently, reducing the concentration of elevated ROS is an important therapeutic goal. Recently, inhibition of ROS production has emerged as a promising approach for treating various diseases due to their diverse impact on cellular physiology. This section aims to examine the most notable inhibitors of ROS production and assess their potential as promising research tools and drug candidates (Chocry & Leloup, 2020; Gurevich & Gurevich, 2014). Over the past two decades, research efforts have focused on developing specific ROS inhibitors, resulting in a wide range of compounds with varying levels of specificity. These inhibitors can be classified into non‐selective inhibitors, selective inhibitors, and inhibitors with an unknown mode of action/specificity. The following sub‐sections will provide a brief overview of these inhibitors.

### 5.1 Non‐selective ROS inhibitors

The first molecules to combat oxidative stress were antioxidants and ROS scavengers, such as polyphenols, vitamins C and E, and N‐acetylcysteine. These molecules were found to have positive effects against various diseases (Abraham et al., 2019; Hall et al., 2019; Lee & Han, 2018; Liu, Liu, Han, et al., 2021; Liu, Liu, Lu, & Zhao, 2021; Pérez‐Torres et al., 2017; Su, Shen, et al., 2019; Su, Zhang, et al., 2019; Zhou et al., 2019). Additionally, the body has built‐in ROS inhibitors and scavengers, such as glutathione (GSH) and pyruvate. GSH, a cysteine‐based tripeptide found in all cell types, primarily exists in the cytosol and the mitochondria, which helps protect against oxidative stress conditions (Calabrese et al., 2017). Depleting GSH can lead to changes in the permeability of the mitochondrial membrane, resulting in a loss of membrane potential and increased ROS production (Marí et al., 2020). Hydrogen peroxide (H_2_O_2_) is reduced with the oxidation of glutathione by GSH‐peroxidase, creating glutathione disulfide and thus exhibiting its ROS‐scavenging properties (Espinosa‐Diez et al., 2015). Likewise, pyruvate, a product of glycolysis, has also been found to act as an antioxidant for H_2_O_2_ through direct and indirect means (Ramos‐Ibeas et al., 2017). The direct method involves the non‐enzymatic decarboxylation of pyruvate to acetate, which reduces H_2_O_2_ to H_2_O. At the same time, the indirect method includes pyruvate metabolism, which leads to reduced GSH through NADPH (Liu et al., 2018).

β‐Carotene is a predominant member of the carotenoid family and is the primary source of provitamin A. Additionally, it can regulate ROS levels through its antioxidant properties. This can be accomplished by direct scavenging of singlet oxygen or by inhibiting lipid peroxidation (Nishino et al., 2017). The interaction between β‐carotene and singlet oxygen can result in the formation of various endoperoxides and epoxides during methyl linoleate chlorophyll‐sensitized photooxidation or the production of cyclic mono‐endoperoxides and di‐endoperoxides in bacteriopheophytin‐sensitized systems. While β‐carotene is known for its ability to scavenge and neutralize ROS, it has also been found to have pro‐oxidant effects at high concentrations (Kawata et al., 2018; Nishino et al., 2017). Similarly, nicotinamide (vitamin B3) is a naturally occurring compound that converts to nicotinamide adenine dinucleotide (NAD+) (Choi et al., 2015). Research findings showed that this compound affects the activity of specific proteins, such as SIRT1, which can regulate autophagy and apoptosis involved in cell survival (Sikora et al., 2008). Additionally, nicotinamide has a direct scavenging effect on ROS. It can decrease intracellular levels of ROS by reducing the electron transport chain and decreasing the production of the mitochondrial permeability transition pore, increasing the membrane potential (Song et al., 2017). Nicotinamide riboside (NR) is a compound that can increase levels of NAD+ in the body. NAD+ is essential in regulating oxidative stress, a type of cell damage caused by harmful molecules called free radicals. Studies have shown that NR can reduce oxidative stress, inflammation, and cell death in the lungs and heart. This leads to less damage to these organs and a lower risk of death (Hong et al., 2018).

Melatonin is a hormone naturally produced by the body's pineal gland. It plays a role in various bodily functions such as immune system regulation, regulating sleep patterns, and heart function (Hacışevki & Baba, 2018). Melatonin is also a “suicidal antioxidant” because it neutralizes harmful free radicals by donating an electron to them, which causes their oxidation (Chainy & Sahoo, 2020; Martinez et al., 2005). According to a study conducted on ischemia/reperfusion (I/R), injured aged Wistar rats administrated nicotinamide mononucleotide and melatonin alone and in combination revealed that all treatments reduced the levels of oxidative stress and harmful ROS produced by the mitochondria. Moreover, they improved the function of the mitochondria, including their membrane potential and the balance of NAD+ and NADH. Furthermore, the combined therapy of melatonin and nicotinamide mononucleotide (NMN) was more effective than either treatment alone in reducing mitochondrial ROS and oxidative stress and improving mitochondrial membrane potential. This suggests that combining melatonin and NMN could be a promising strategy for protecting the I/R‐damaged heart. Additionally, the restoration of mitochondrial function is a pivotal contributor to heart protection (Hosseini et al., 2020).

Edaravone is a small, fat‐soluble compound that can cross the blood–brain barrier and has antioxidant properties. It can scavenge free radicals and inhibit lipid peroxidation. It additionally reduces damage caused by oxidative stress and may activate the Nrf2/ARE pathway (Ismail et al., 2020). In Japan, Edaravone is the only neuroprotective agent for acute ischemic stroke (Cha & Kim, 2022). It neutralizes excessive ROS levels, which helps prevent brain damage (Matsumoto et al., 2018). Furthermore, studies involving animal models showed that Edaravone could protect against oxidative damage to the retina and cell death caused by oxidative stress (Inokuchi et al., 2009). N‐Acetyl cysteine (NAC) is a prodrug form of cysteine that has been widely used in in vitro and in vivo studies as a free radical scavenger. It is also known as an acetylated precursor of GSH (Ezeriņa et al., 2018; Zhang, Gan, et al., 2011; Zhang, Lau, & Monks, 2011). NAC has scavenging activity through its thiol and by converting to potential ROS‐scavenging sulfane species through enzymes such as 3‐mercaptopyruvate sulfurtransferase and sulfide‐quinone oxidoreductase. However, NAC has been observed to have a damaging effect on cellular viability through modulation of the redox state of specific kinases, resulting in the activation of a cell‐cycle‐dependent kinase inhibitor. This suggests that NAC is not only an ROS scavenger but also a compound that can have other biological effects (Ezeriņa et al., 2018; Kim, Choi, et al., 2001; Kim, Rhim, et al., 2001; Zhang, Gan, et al., 2011; Zhang, Lau, & Monks, 2011). Likewise, nitrones are a group of antioxidant compounds that have neuroprotective properties and could be used in the treatment of conditions such as traumatic injuries, ischemic strokes, and neurodegenerative diseases. Studies have shown promising results in their ability to protect brain cells and prevent ROS‐induced damage in these conditions (Cancela et al., 2020; Clausen et al., 2008; Marco‐Contelles, 2020; Marklund et al., 2001; Nash et al., 2018). NXY‐059, TBN, thiadiazole 4a, QN23, and QN6 are some examples of nitrones effective against neurodegenerative ailments and strokes (Chioua et al., 2019; Shuaib et al., 2007).

### 5.2 ROS inhibitors targeting specific sites

Unlike scavenging inhibitors discussed above, ROS inhibitors that target particular sites have a specific mechanism of action to inhibit the production of ROS without the adverse effects of reducing their levels throughout the organism, which may alter the helpful effects associated with them (Brieger et al., 2012). Over the past decade, a group of inhibitors for the NOX enzyme have been developed and claimed to be specific for certain isoforms. However, not all of these inhibitors have been proven to have selectivity for a single isoform or to specifically target NOX over other oxidases.

GenKyoTex has developed two dual inhibitors, GKT136901 and GKT137831, specifically designed to inhibit the NOX1 and NOX4 isoforms. Both inhibitors are highly potent in inhibiting NOX1 and NOX4, with reported IC_50_ values of 0.14 and 0.11 μM for GKT137831 and 0.160 and 0.165 μM for GKT136901, respectively (Aoyama et al., 2012; Sedeek et al., 2010). Additionally, GKT136901 and GKT137831 have been found to inhibit (IC_50_: 0.4 μM) NOX5 activity (Musset et al., 2012). Moreover, GKT136901 and GKT137831 were found not to affect xanthine oxidase and several other unrelated targets, such as G‐coupled protein receptors, kinases, and channels for calcium, potassium, and chloride ions (Laleu et al., 2010). These inhibitors do not affect other enzymes that produce ROS. Nevertheless, GKT136901 has been found as a potential scavenger of peroxynitrite (Schildknecht et al., 2014).

In vivo, GKT136901 has been found to decrease angiogenesis and reduce tumor growth via inhibition of NOX1 (Garrido‐Urbani et al., 2011). In addition, GKT136901 has been found to disrupt the function of DUOX‐1 and DUOX‐2, whereas GKT137831 has also been shown to have an effect against NOX2, but it is less effective against NOX2 than it is against NOX1 and NOX4 (Aoyama et al., 2012; Augsburger et al., 2019). Both inhibitors can be taken orally and effectively prevent hypertension, oxidative stress conditions, and albuminuria, as experimented with in various animal models (Sedeek et al., 2010; Zeng et al., 2019). GKT137831, orally bioavailable, is being tested in phase‐II trials to treat primary biliary cholangitis, Type‐I diabetes, and pulmonary fibrosis. Moreover, GenKyoTex has also developed GKT771, another specific and potential NOX1 inhibitor. GKT771 is designed to inhibit NOX1; it does not inhibit other forms of NOX, XO, or glucose oxidases with similar potentiality and does not function as an ROS scavenger. Furthermore, it was found to inhibit the growth of colon cancer in mice by boosting immunity and suppressing angiogenesis. This inhibitor might be a potent suppressor of tumor cells subject to proper immune system functionality (Stalin et al., 2019).

Similarly, in 2016, the Ewha Woman's University of Seoul developed APX‐115 (Ewha‐18,278) as a novel pan‐NOX inhibitor. This substance has been shown to inhibit the activity of NOX1 (IC_50_: 1.08 μM), NOX2 (IC_50_: 0.57 μM), and NOX4 (IC_50_: 0.63 μM) enzymes without affecting the activity of other enzymes such as xanthine and glucose oxidases or scavenging ROS (Cha et al., 2017; Joo et al., 2016). This inhibitor is available for oral consumption and has demonstrated significant pharmacokinetics (Lee et al., 2019). Its effectiveness is comparable to that of losartan, a reno‐protective medication employed for diabetic patients. In addition, this compound helps prevent kidney injury and protects the functionality of mitochondria from damage due to lipid peroxidation. The substance APX‐115 also has protective effects against renal injury caused by Type‐I and II diabetes in various models (Dorotea et al., 2018; Kwon et al., 2017). Furthermore, it has potential therapeutic benefits in the treatment of osteoporosis (Joo et al., 2016).

Vasopharm developed VAS2870 (also known as NOX inhibitor‐III), a triazolo pyrimidine derivative. Research findings showed that it significantly inhibits the activity of NADPH oxidase induced by platelet‐derived growth factor in rat vascular smooth muscle cells (ten Freyhaus et al., 2006). Moreover, it does not exhibit antioxidant properties or affect xanthine oxidase activity. In human neutrophil lysates, this substance was found to inhibit (IC_50_: 10.6 μM) the activity of NOX2 (Gatto Jr et al., 2013) and NOX1, 2, 4, and 5 (Dao et al., 2020). Notably, it only inhibits NOX2 activity when added before the formation of the enzyme complex and has no effect once the enzyme complex is assembled. This suggests that the inhibitory action of VAS2870 against NOX2 is due to the prevention of enzymatic complex formation (Altenhöfer et al., 2012). Additionally, VAS2870 may have potential therapeutic applications in various pathological conditions such as thrombosis, tumors, and cardiovascular and neurodegenerative diseases (Kleinschnitz et al., 2010; Liu et al., 2017; Sancho & Fabregat, 2011). Furthermore, Vasopharm developed VAS3947, also known as NOX inhibitor‐VIII, by modifying VAS2870 to increase its solubility. VAS3947 specifically inhibits NOX activity and does not affect xanthine oxidase, or NOS. In this respect, studies in 2010 revealed that VAS3947 could inhibit the isoforms NOX1 (IC_50_: 12 μM), NOX2 (IC_50_: 2 μM), and NOX4 (IC_50_: 13 μM) (Wind et al., 2010).

In 2015, researchers developed GLX351322 as a highly effective inhibitor of the enzyme NOX4, with an IC_50_ value of 5 μM. It also reduces NOX2 activity in hPBMC cells to a lesser extent, as the reported IC_50_ value was 40 μM. The inhibitor enhanced the β‐cells' functionality and prevented their death from hyperglycemia‐induced oxidative stress (Anvari et al., 2015; Augsburger et al., 2019). More recently, GLX481372 and GLX7013114 were the two NOX4 inhibitors developed. GLX481372 was found to have inhibitory activity against both NOX4 and NOX5 with IC_50_ values of 0.68 and 0.57 μM, respectively, while they reduced the activity of NOX1 and NOX2 to a lesser extent (IC_50_: 7 and 16 μM, respectively) (Wang, Elksnis, et al., 2018; Wang, Xiao, et al., 2018). Likewise, GLX7013114 specifically targets NOX4 with high potentiality and has no documented inhibitory action against other NOX isoforms or other enzymes. An in vitro investigation has suggested that it may protect against cellular death caused by cytokines or high glucose and palmitate levels. Moreover, it has an exceptional inhibitory potential against NOX4 owing to its enhanced specificity (Wang, Elksnis, et al., 2018; Wang, Xiao, et al., 2018).

NOS31, another selective inhibitor derived from *Streptomyces* sp., inhibits the growth of cancer cells in various types of cancer associated with increased expression of NOX1, including stomach and colon cancer cells. NOS31 and its analog NOS35 were isolated in 2018 from *Streptomyces* sp. bacteria. NOS31 is highly selective and only affects cancer cells with high levels of NOX1, and neither interferes with XO nor acts as a scavenger of hydrogen peroxide. Inhibiting NOX2, NOX3, NOX4, and NOX5 with this inhibitor is 14 times weaker than NOX1. NOS31 has an IC_50_ value of 2.0 μM for NOX1 and 28.7 μM for NOX4. No inhibition was observed in the experimented concentration range for NOX2, NOX3, and NOX5. Furthermore, NOS31 may be helpful as a tool compound, but more selectivity testing is needed before it can be widely adopted (Yamamoto et al., 2018). Selective NOX2 inhibitors, such as CPP11G and CPP11H, may target NOX2 specifically (Cifuentes‐Pagano et al., 2013). These compounds, which are inactive against other NOX isoforms and do not possess any interaction with ROS, hinder the movement of a protein called p47phox from the cytosol to the plasma membrane, preventing interactions that lead to inflammation and vascular dysfunction (Li et al., 2019). However, more research is needed as their potency is low and their profiling is limited.

NOX2ds‐tat (NOX2) is a peptide inhibitor developed in 2011 by Patrick Pagano's group. It works by binding to the p47phox subunit, preventing the assembly and activation of the NOX2 complex (IC_50_: 0.74 μM) (Csányi et al., 2011). NOXA1ds (NOX1) is another peptide inhibitor that the same group developed in 2013. It is specific to NOX1 and disrupts the association of NOX1‐NOXA1; it significantly inhibits the activity of NOX1 for whole HT29 cells (IC_50_: 100 nM) and cell lysates (IC_50_: 19 nM). NOXA1ds does not bind or inhibit NOX2 and NOX4, nor does it affect NOX5 and xanthine oxidase activities (Ranayhossaini et al., 2013). It has been used to examine the effect of NOX1 on endothelial cell proliferation, migration, and hypertension (Csányi et al., 2011; Neves et al., 2018; Ranayhossaini et al., 2013). On the other hand, monoamine oxidase (MAO) inhibitors, such as iproniazid, have been widely used as antidepressants. Iproniazid, initially developed as an antituberculosis agent, was the first drug used as an antidepressant through the inhibition of the MAO enzyme. The “cheese effect” is a side effect caused by the buildup of dietary tyramine, commonly found in cheese, which can lead to an increased release of noradrenaline and a rise in blood pressure when taking MAO inhibitors (Manzoor & Hoda, 2020).

MAO inhibitors are currently used to treat neurodegenerative diseases by inhibiting the enzymes responsible for neurotransmitter degradation, like serotonin and dopamine. MAO‐A and MAO‐B enzymes are involved in degrading neurotransmitters, such as dopamine and serotonin, by catalyzing the oxidative deamination of biogenic amines and producing hydrogen peroxide or ammonia as byproducts (Youdim, 2018). Research findings indicated that several MAO‐B inhibitors, such as selegiline and rasagiline, could slow the progression of PD (Binde et al., 2018; Hauser et al., 2017). Selegiline, a selective MAO‐B inhibitor, has been found to improve life expectancy in patients suffering from PD when combined with L‐DOPA; it is now widely used as a treatment option for the disease (Filip & Kolibas, 1999; Guay, 2006). In addition to their use in treating PD, MAO‐B inhibitors have also been studied for their potential in treating other conditions, such as AD and cancer. Their effects on vascular function in diabetes have also been investigated (Lighezan et al., 2016; Tábi et al., 2020). On the other hand, rasagiline, an irreversible inhibitor of the MAO‐B enzyme, was approved for use as a treatment option for PD in the EU in 2005 (Lecht et al., 2007). It has a higher inhibitory potency for MAO‐B than MAO‐A and binds covalently to the FAD moiety of MAO‐B (Guay, 2006). Moreover, ladostigil (TV3326), a propargylamine derivative and MAO‐A and MAO‐B inhibitor, is promising as a neuroprotective agent and is used in managing AD and PD (Xu et al., 2018).

### 5.3 Inhibitors with an unknown mode of action/specificity

This section deals with compounds that do not interfere with ROS through direct chemical scavenging or interaction with ROS production systems discussed earlier. However, many of these compounds have an unknown mode of action/specificity. In this context, perhexiline is a medication used to prevent angina and is approved for use in Australia and New Zealand. Studies showed that it alters cardiac metabolism and prevents ROS production by inhibiting (IC_50_: 2.3 μM) a specific enzyme called NOX2 (Gatto Jr et al., 2013). The exact mechanism of how it inhibits is not entirely clear, but it has been determined that it does not affect the assembly of NOX2 and is not a scavenger of superoxide. However, it has not yet been studied on other forms of NOX, such as NOX1, NOX3, NOX4, and NOX5. Additionally, it is important to note that it needs to be metabolized by an enzyme called CYP2D6, and it has side effects such as nausea, liver damage, and nerve damage (Gehmlich et al., 2015). Shionogi 1 and 2 are compounds that have been reported to inhibit the enzyme NOX2, but they do not do so in a direct manner. Instead, they inhibit a protein called protein kinase C beta II (PKCbeta II), which prevents it from performing its role in the movement of a protein called p47phox. As a result, these compounds specifically target NOX2, making them useful for specific research. Shionogi 1 and 2 have been reported to have IC_50_ values of 56 nM and 99 nM, respectively, for NOX2 inhibition (Gatto Jr et al., 2013). In addition, they were also found to affect xanthine oxidase activity. However, it is important to remember that their mode of action does not directly involve binding to and inhibiting NOX2 (Gatto Jr et al., 2013).

## 6 CONCLUSIONS

In summary, the concept of ROS as a two‐edged sword in health and pathology has gained significant importance, despite the unclear mechanisms involved. Levels of metabolic ROS in diverse physiological processes are strongly regulated and have a vital role as a signaling pathway, contributing to cellular homeostasis. In acute and chronic oxidative stress conditions, excessive ROS production and diminished antioxidative activities are related to inflammation, cellular dysfunctionality, senescence, diseases, and death. Data obtained in this review reveal that ROS elevation disturbs redox homeostasis and leads to the induction of oxidative stress conditions. In addition, ROS impairs the functionality of vital biomolecules and organelles like DNA, proteins, and lipids, leading to various diseases such as carcinogenesis, diabetes, neurodegeneration, aging, AD, PD, and prion disease. Disruption of redox equilibrium owing to increased aggregation or reduction of ROS has affected various signaling pathways, resulting in cellular dysfunctionality and the progression of different diseases. Consequently, the mechanisms involved in ROS that regulate redox signaling pathways aid in providing associated targets for developing efficient therapeutic strategies. Nevertheless, interactions among various ROS‐sensitive signaling pathways are yet to be investigated in detail. Along this line, several natural and synthetic antioxidants have been studied for their ROS‐scavenging activity. However, further studies are needed to understand synthetic and natural compounds' potential in inhibiting cellular injury caused by ROS and in treating various associated diseases.

## AUTHOR CONTRIBUTIONS

**Abdur Rauf:** Conceptualization (equal); data curation (equal); formal analysis (equal); investigation (equal); visualization (equal). **Anees Ahmed Khalil:** Conceptualization (equal); formal analysis (equal); investigation (equal); methodology (equal); visualization (equal); writing – original draft (equal). **Samir Awadallah:** Investigation (equal); methodology (equal); visualization (equal). **Shahid Ali Khan:** Supervision (equal); validation (equal). **Tareq Abu‐Izneid:** Data curation (equal); resources (equal); validation (equal); visualization (equal). **Muhammad Kamran:** Validation (equal); visualization (equal); writing – review and editing (equal). **Hassan A. Hemeg:** Resources (equal); validation (equal); writing – review and editing (equal). **Mohammad S. Mubarak:** Resources (equal); supervision (equal); validation (equal); writing – review and editing (equal). **Ahood Khalid:** Resources (equal); validation (equal); visualization (equal); writing – review and editing (equal). **Polrat Wilairatana:** Conceptualization (equal); investigation (equal); resources (equal); supervision (equal); validation (equal); writing – review and editing (equal).

## CONFLICT OF INTEREST STATEMENT

The authors declare no conflict of interest.

## Data Availability

The dataset supporting the conclusions of this article is included within the report.
